# Longitudinal monitoring of handgrip strength in rheumatoid arthritis: a window into for disease activity—a systematic review with meta-analysis

**DOI:** 10.1136/bmjsem-2025-002617

**Published:** 2025-11-21

**Authors:** Rafaela Cavalheiro do Espirito Santo, Leonardo Peterson dos Santos, Geiziane Melo, Viney Dubey, Cesar Agostinis-Sobrinho

**Affiliations:** 1 Health Research and Innovation Science Centre, Klaipeda University, Klaipeda, Lithuania; 2 Post Graduate Program in Medicine: Medical Sciences, UFRGS, Porto Alegre, Brazil

**Keywords:** Aging, Disability, Elderly people, Health promotion, Measurement

## Abstract

**Objective:**

Handgrip strength (HGS) is a simple, non-invasive measure associated with disability, frailty and disease activity in chronic conditions such as rheumatoid arthritis (RA). However, longitudinal changes in HGS and their implications in RA remain underexplored. This study aimed to systematically review changes in HGS over time and its associations with disease status and follow-up duration in patients with RA.

**Design:**

Systematic review and meta-analysis.

**Data sources:**

A search of PUBMED, EMBASE and WEB OF SCIENCE were searched for cohort studies up to July 2025.

**Eligibility criteria:**

Studies including RA patients, assessing HGS and employing an observational design.

**Results:**

From 4301 studies (including 737 identified through citation tracking), 27 met the inclusion criteria, comprising 2742 individuals (mostly women (1784; 65.1%)), aged 19–87 years, with disease duration ranging from 2 months to 47 years. Participants generally had low to moderate disease activity and moderate to severe physical disability. Overall, HGS slightly increased over time (standardised mean difference; SMD 0.25; 95% CI 0.07 to 0.43). Greater improvements were observed in early RA (SMD 0.46; 95% CI 0.30 to 0.61), while no significant changes were found in established RA. HGS increased in patients followed for ≤1 year (SMD 0.25; 95% CI 0.07 to 0.43) and >1–5 years (SMD 0.43; 95% CI 0.05 to 0.81), but not beyond 5 years.

**Conclusion:**

Patients with early RA tend to improve HGS over time, whereas those with longer disease duration show stable strength levels. HGS may serve as a useful marker for monitoring function and guiding personalised care in RA.

**PROSPERO registration number:**

CRD42023473416.

**WHAT IS ALREADY KNOWN**

- Rheumatoid arthritis (RA) is a systemic autoimmune disease that causes inflammation, leading to joint and extra-articular damage, including muscle loss and reduced strength, particularly handgrip strength.
- Cross-sectional studies provide evidence that 20.4% of patients with RA exhibited low handgrip strength.
- Patients with new or active RA have lower handgrip strength compared with those in remission and healthy controls.

**WHAT ARE THE NEW FINDINGS**

- Our meta-analysis demonstrated a slight increase in handgrip strength over time in RA patients.
- Additionally, patients with early RA showed an increase in handgrip strength, whereas those with established RA did not exhibit any change in handgrip strength over time.
- Patients followed for up to 1 year, as well as those followed for more than 1 year but less than 5 years, showed an increase in handgrip strength; however, patients followed for more than 5 years did not demonstrate any changes in handgrip strength.

## Introduction

 Rheumatoid arthritis (RA) is a systemic autoimmune disease characterised by inflammation, leading to both joint and extra-articular manifestations.^1 2^ Overall, the behavioural, biological and physiological changes are damaging to several tissues,^3^ including muscle tissue in these patients. Consequently, these changes lead to reductions in muscle mass and muscle strength, especially handgrip strength (HGS), regardless of age. Cross-sectional studies provide evidence that 20.4% of patients with RA exhibited low HGS.^4^ Additionally, patients with new or active RA have lower HGS compared with those in remission and healthy controls.^5 6^

Despite clear cross-sectional evidence, temporal changes in HGS among patients with RA, as well as its associations with related factors, remain poorly defined. There is only one systematic review based on cross-sectional studies,^7^ published in 2010, describing the patterns of HGS loss with age in the general population and in patients with RA. The authors reported that in the general population, mean HGS decreased from 45.5 kg to 23.2 kg in males and from 27.1 kg to 12.8 kg in females between the ages of 25 years and 95 years. Conversely, although HGS remained stable in the treated RA population between the ages of 35 years and 65 years, it was lower compared with the healthy population. Also, RA disease duration was significantly associated with a decline in handgrip overtime, with an annual rate of loss of 0.34 kg at age 55.6 years (95% CI −0.58 to −0.10). By focusing on cross-sectional studies, Bernanke *et al*^7^ were able to provide insights into how handgrip strength varies across different age groups within the RA population.

However, to date, no systematic review has examined longitudinal changes in HGS among patients with RA, nor have causal associations been explored. This gap limits the understanding of how muscle function evolves over time and its potential relationship with disease progression or activity. This limits the understanding of how muscle function changes over time and how it may relate to disease progression or disease activity levels. Addressing these gaps carries important clinical implications. first, understanding how muscle function changes over time could facilitate the early identification of functional decline, enabling timely and targeted interventions. Should causal associations between reduced HGS and disease activity or progression be established, this measure could serve as a simple, non-invasive and cost-effective biomarker to support clinical decision-making and disease monitoring. Moreover, longitudinal insights may enhance patient stratification, contributing to more personalised treatment strategies and the prevention of sarcopenia-related complications. Ultimately, incorporating routine assessments of HGS into clinical practice could strengthen multidisciplinary care approaches and inform future guidelines aimed at preserving physical function and improving long-term outcomes in patients with RA.

Therefore, the aim of this study was to systematically review longitudinal changes in HGS and its associations with disease status and follow-up duration in patients with RA. Our primary hypothesis posited a decline in HGS over time among patients with RA. Furthermore, we hypothesised that disease status and follow-up duration would be associated with this decline.

## Methods

This systematic review was conducted in accordance with Preferred Reporting Items for Systematic Reviews and Meta-Analyses (PRISMA) guidelines^8^ (online supplemental data 1) after registering the protocol on the PROSPERO platform (CRD42023473416; https://www.crd.york.ac.uk/PROSPERO/view/CRD42023473416). No amendments were made to the protocol following registration.

### Eligibility criteria

Studies were required to meet the following criteria related to Participants, Exposure, Comparison, Outcomes and Study (PECOs criteria):

P— rheumatoid arthritis;

E —none;

C—none;

O— handgrip strength;

S—observational studies.

The inclusion criteria were as follows:

- Observational studies involving patients with RA that quantitatively assessed HGS using a dynamometer.
- Studies that described HGS at follow-up(s).
- Publications in English.

The exclusion criteria were as follows:

- Studies conducted in experimental models, randomised clinical trials (eg, changes in grip strength after exercise training or dietary supplementation) or cross-sectional studies as well as reviews.
- Studies focused on diseases other than RA or including data from patients under 18 years old (paediatric patients).
- Publications based on the same dataset (two or more studies analysed data from the same population), only one of them was included in the review. This approach avoids duplicate or overlapping data, which could skew the results and introduce bias.
- Studies published in languages other than English.

### Information sources

The bibliographic databases PubMed, EMBASE and Web of Science were systematically searched up to 17 September 2024. No restrictions were applied regarding language or publication date. In July 2025, forward and backward citation tracking was performed on the references cited in the studies included in this systematic review to identify additional relevant articles. To ensure transparency and reproducibility, this dataset has been deposited in the Open Science Framework (OSF) and is publicly accessible at DOI: 10.17605/OSF.IO/B5P39.

### Search strategy

The search strategy was developed with the support of an experienced. It was based on Medical Subject Headings, subject-specific terms and relevant synonyms. A comprehensive search strategy, tailored to each database, was employed.

- Pubmed- (Arthritis, Rheumatoid[mh] OR Rheumatoid Arthritis[tiab]) AND (Muscle Strength[mh] OR Muscle Strength[tiab] OR Arthrogenic Muscle Inhibition*[tiab] OR Hand Strength[tiab] OR Grip[tiab] OR Grips[tiab] OR Grasp[tiab] OR Grasps[tiab] OR Pinch Strength[tiab]);
- Embase - ('rheumatoid arthritis'/exp OR 'Rheumatoid Arthritis':ti,ab,kw) AND ('muscle strength'/exp OR ('Muscle Strength' OR 'Arthrogenic Muscle Inhibition*' OR 'Hand Strength' OR Grip OR Grips OR Grasp OR Grasps OR 'Pinch Strength'):ti,ab,kw) AND [embase]/lim NOT ([embase]/lim AND [medline]/lim);
- Web of Science- TS=("Rheumatoid Arthritis") AND TS=("Muscle Strength" OR "Arthrogenic Muscle Inhibition*" OR "Hand Strength" OR Grip OR Grips OR Grasp OR Grasps OR "Pinch Strength");

### Selection process

Title, abstract and full-text screening were conducted independently and in duplicate by two reviewers (RES and LdS), using the Rayyan platform,^9^ a web-based tool designed to support blinded screening in systematic reviews. Any discrepancies between the two reviewers were resolved through discussion. If full-text articles were not accessible through databases or open-access sources, we attempted to contact the corresponding authors via email or ResearchGate. Each author was contacted up to two times within a period of 4 weeks. When no response was received and the full text could not be obtained, the study was excluded from the review. The study selection process is detailed in online supplemental data 2, while the outcomes of the forward and backward citation tracking are presented in online supplemental data 3. To ensure transparency and reproducibility, this dataset has been deposited in the OSF and is publicly accessible at DOI: 10.17605/OSF.IO/B5P39.

### Data collection process

The data collection process was conducted by one reviewer (RES) and reviewed by another reviewer (LdS) using Microsoft Excel. When any data were missing, the authors of the original studies were contacted to obtain or confirm the information. No automated tools were used to assist in the data extraction process.

### Data item

From each included study, the following data items were systematically extracted: first author’s name, year of publication, country of origin, duration of follow-up, total sample size, sex distribution, central tendency and dispersion measures of age (mean or median), indicators of disease activity, measures of physical function, pharmacological treatment status, type of device or protocol used for HGS assessment, descriptive statistics related to HGS and any reported associations between HGS and clinical outcomes. When studies stratified the sample into groups, data extraction was prioritised from groups that had not undergone surgical interventions, included the largest sample size, had not initiated pharmacological treatment or had maintained their habitual routines. This strategy was adopted to maximise comparability across studies and minimise heterogeneity.

Quantitative data were extracted as reported in the original articles, including sample size, percentages, means and SD or medians and IQRs. When only IQRs were provided, values were converted to means and SD using established estimation methods described in the literature^10 11^ to enable inclusion in the meta-analysis. In cases where data were not provided, the respective authors were contacted to request the missing information.

### Methodological quality assessment

The methodological quality assessment was conducted independently by two reviewers independent (RES and VD) in pairs. Disagreements were resolved through discussion with a third reviewer (GM). The Newcastle–Ottawa Quality Assessment Scale for cohort studies was used to assess the methodological quality of the articles included. Each study was evaluated based on eight items categorised into three groups: selection, comparability and outcome. Thresholds for converting the Newcastle–Ottawa scales to the Agency for Healthcare Research and Quality standards were as follows^12 13^:

- Good quality (three or four stars in the selection domain AND one or two stars in the comparability domain AND two or three stars in the outcome/exposure domain).
- Fair quality (two stars or three in the selection domain AND one or two stars in the comparability domain AND two or three stars in the outcome/exposure domain);
- Poor quality (zero or one star in the selection domain OR zero stars in the comparability domain OR zero or one star in the outcome/exposure domain).

### Study risk of bias assessment

Assessment of reporting bias and certainty of evidence not conducted, as the primary objective of this systematic review was to characterise longitudinal patterns of change in HGS rather than to evaluate the effectiveness of interventions.

### Statistical analysis

The meta-analysis was conducted using Review Manager V.5.4.1 (RevMan), software developed by The Cochrane Collaboration in 2020. First, the authors analysed overall changes in HGS over time in studies that included both sexes. Second, the authors analysed changes in HGS over time in patients with early RA and established RA (early RA was defined as disease duration <1 year, and established RA as disease duration >1 year). Third, the authors analysed changes in HGS over time according to follow-up time: until 1 year, from 1 to 5 years and above 5 years. Fourth, the authors analysed changes in HGS in women and men.

The meta-analysis was conducted using mean and standardised mean from each study. All outcome measures were continuous variables. A random-effects model with standardised mean difference (SMD) or median difference (MD) was employed when appropriate. The MD was used when the included studies reported outcomes using the same assessment scale. In contrast, the SMD was applied when the same outcome was assessed across studies but measured using different scales.^14^ The SMD was obtained by dividing the difference in means between groups by the pooled SD. The formulas used for these calculations are described in detail in previous publications.^10 15^

The 95% CIs were calculated, and the heterogeneity of the included studies was assessed using the inconsistency test (I^2^). We considered values of approximately 25%, 50% and 75% to indicate low, moderate and high inconsistency, respectively.^13^ To explore potential sources of heterogeneity, subgroup analyses were conducted based on disease duration, sex and, when possible, the unit of measurement used to assess HGS. We inspected funnel plots to verify whether publication bias. Finally, to facilitate the interpretation of the SMD and to highlight its clinical relevance, we calculated the absolute mean difference by multiplying the SMD by an estimate of the SD associated with each measurement unit.^14^ This adjustment was obtained by calculating the weighted baseline SD for each instrument, multiplying it by the final SMD of each study, and averaging these values across studies. We aggregated studies reporting outcomes in N and kg, as well as those using pressure-based units (mm Hg and kPa), according to their methodological proximity.^14^ In addition, to further facilitate interpretation, we converted values expressed in N into kilogram-force (kgf) using the standard physical conversion factor (1 n=0.10197 kgf). Conversions and subsequent calculations were performed using the online tool ConvertLIVE (https://convertlive.com/pt/u/converter/newtons/em/quilograma-for%C3%A7a#44.44), accessed 1 October 2025. The converted values were then included in the meta-analysis as absolute mean differences. To ensure transparency and reproducibility, this dataset (online supplemental data 7) has been deposited in the OSF and is publicly accessible at DOI: 10.17605/OSF.IO/B5P39. For all analyses, statistical significance was determined at p<0.05.

## Results

### Study selection

The initial search strategy identified 3564 potentially relevant records, of which 1081 were duplicates and subsequently removed. Following the screening of titles and abstracts, 2346 records were excluded based on the predefined eligibility criteria. Full-text screening was conducted on the remaining articles, resulting in the inclusion of 30 studies. Studies were excluded at this stage due to the absence of HGS data during follow-up, the use of duplicate datasets or the unavailability of full texts despite attempts to contact the corresponding authors via ResearchGate or email, with a waiting period of up to 1 month. Therefore, among these, 20 longitudinal studies were initially selected for this review.^16^,^35^

An updated search was conducted in July 2025 using forward and backward citation tracking. A total of 737 cited references were identified from the previously included studies. Of these, 105 were duplicates and subsequently removed. Following title and abstract screening, 72 studies were excluded as they were already captured in the initial search. An additional 549 studies were excluded based on eligibility criteria. Full-text screening was conducted for the remaining 11 articles, resulting in the inclusion of seven^36^,^42^ additional longitudinal studies that met the predefined criteria.

**Figure 1:**
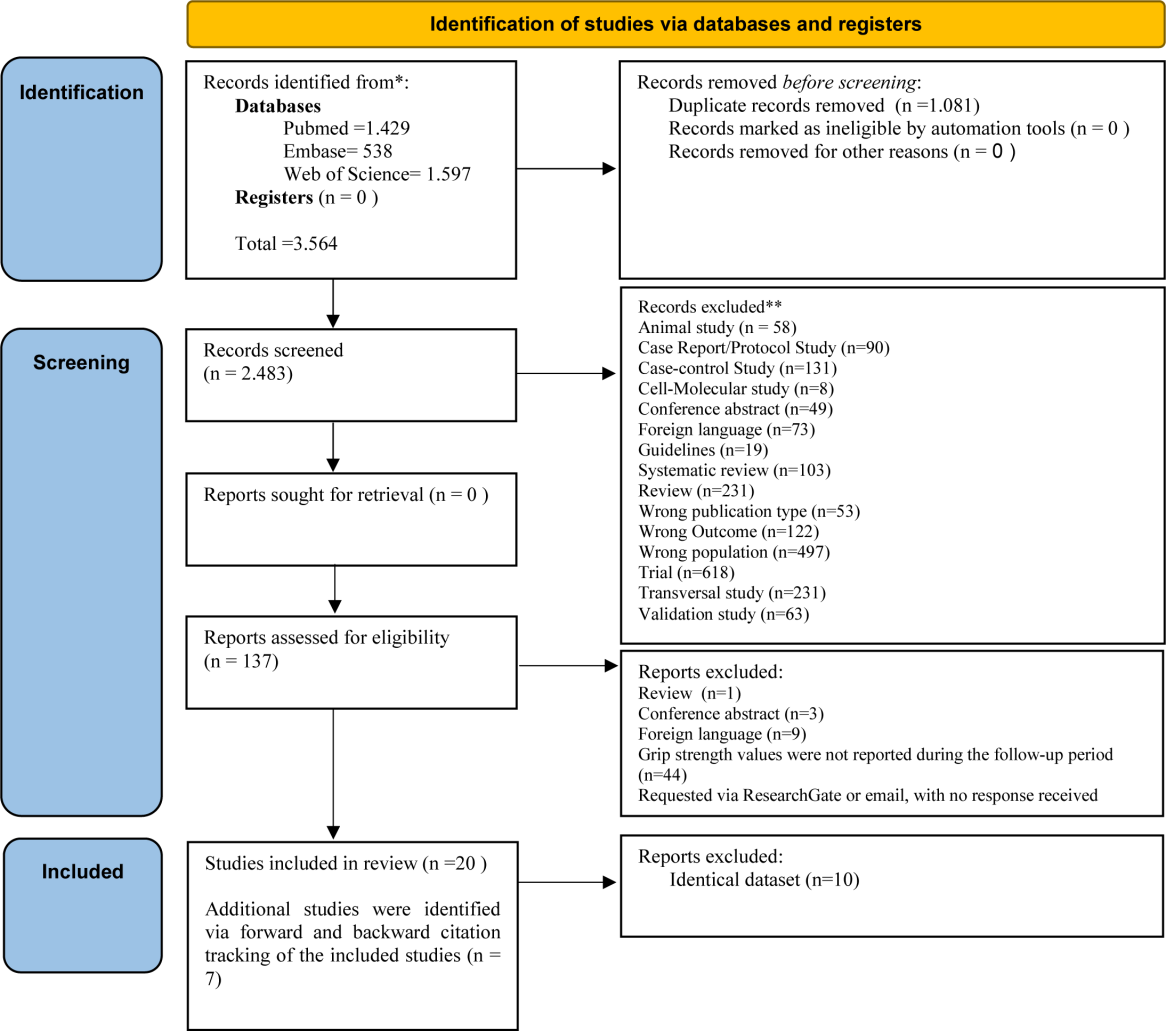
The PRISMA flow diagram of search results and study selection. PRISMA, Preferred Reporting Items for Systematic Reviews and Meta-Analyses.

Therefore, a total of 27 longitudinal studies were included in the final systematic review. The study selection process is illustrated in the PRISMA flow diagram (figure 1).^8^ The lists of studies excluded with the reason are in onlinesupplemental datas 2  3 (forward and backward citation tracking).

### Study characteristics

The studies were published between 1984 and 2024. Their geographical distribution was diverse, comprising six studies conducted in Sweden,^1922^,^24 28 36^ six in the United Kingdom,^27 33 34 38 40 42^ three in the USA,^25 31 41^ and one multicentre study spanning the USA, United Kingdom and Canada.^20^ Additionally, three studies were carried out in the Netherlands,^26 29 30^ and one study each was conducted in Japan,^17^ Brazil,^18^ Italy,^16^ Norway,^21^ Scotland,^39^ Greece,^35^ Israel^37^ and Ireland^32^ (online supplemental data 4). Follow-up durations ranged from 7 months to 10 years, with sample sizes ranging from 22 to 289 patients. The total sample comprised 2742 individuals, the majority of whom were women (1784; 65.1%), aged between 19 and 87 years. Disease durations ranged from 2 months to 61 years, with disease activity levels classified as low to moderate and physical disability levels categorised as moderate to severe. Ten studies^17^,^1922 27 29 32 34 35 37^ reported whether subjects were treated with methotrexate (MTX), 13^1819 21 24^,^27 29 32 36 38 39 41^ with other disease-modifying antirheumatic drugs (DMARDs), 3 with biologic DMARDs (bDMARDs),^17 18 23^ and 11^17^,^1921 24 29 32 34 36 41 42^ with glucocorticoids. Additional clinical features and pharmacological treatments are detailed in onlinesupplemental datas 4  5.

### Methodological quality

Among the 27 studies included, four^16 18 20 36^ received three or four stars in the selection domain, while 23^1719 21^,^35 37^ received only two stars in this domain. Ten studies^18^,^2226 28 33 34 40^ received one star in the comparability domain while 17^1617 23^,^25 27 29^ received 0 stars. In the outcome domain, 15 studies^17^,^2227 30^ received three stars, 10^24^,^2628 29 35^ received two stars and 2^16 23^ received only one star. The predominance of lower scores, particularly in the selection and comparability domains, leads to the conclusion that most studies are of poor quality (16 studies; 59.3%),^1617 24 25 27 29^,^32 35^ followed by those of fair quality (nine studies; 33.3%)^1921^,^23 26 28 33 34 40^ and only a few classified as good quality (two studies; 7.4%).^18 20^ For this reason, it is important to emphasise the need for caution when interpreting the findings. Detailed assessments of methodological quality are provided in online supplemental data 6.

### Results of individual studies

#### Longitudinal changes in HGS

**Figure 2:**
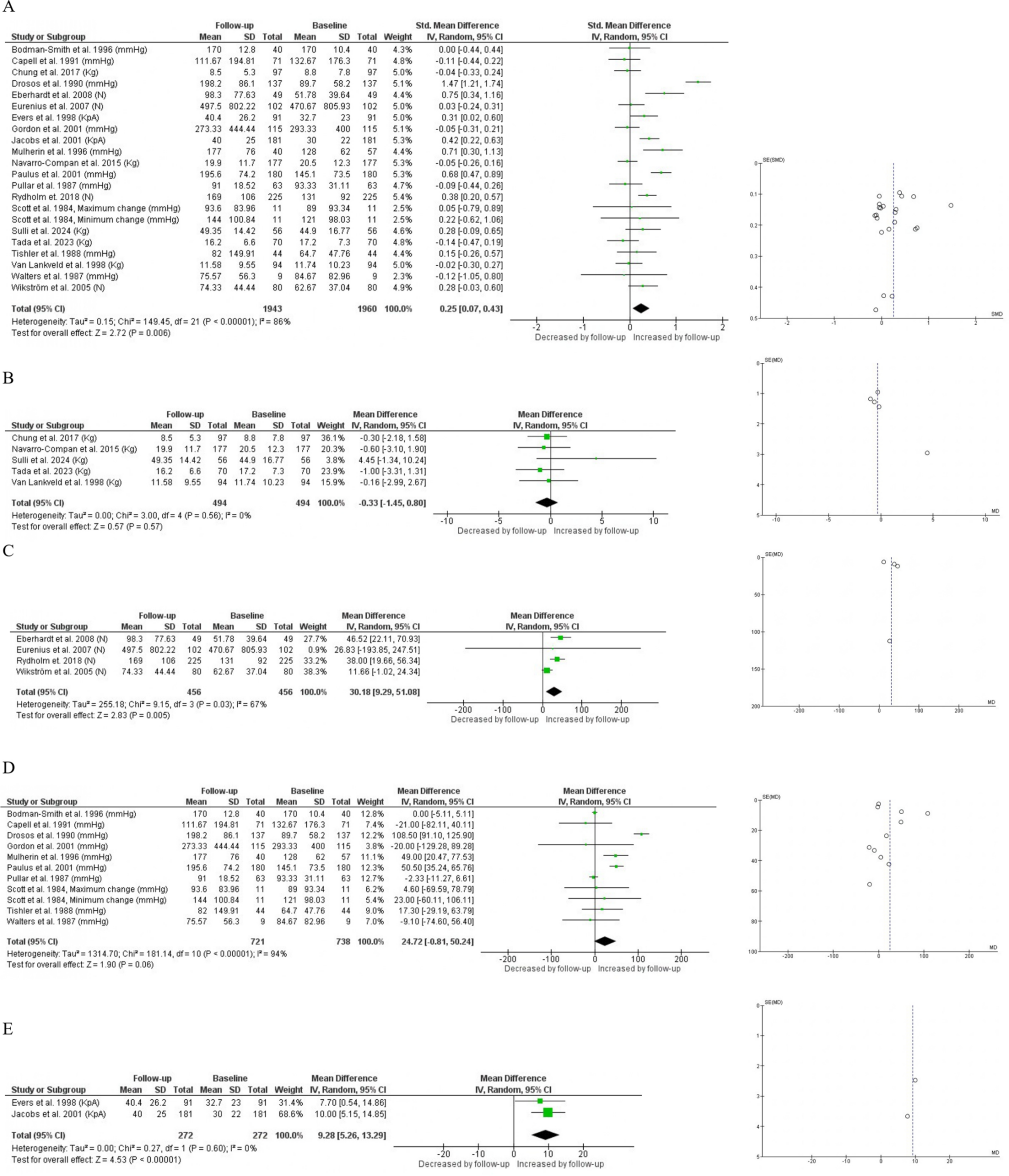
(A) Handgrip strength over time in all studies included. (B) Subgroup analysis in handgrip strength over time for studies that used kg unit. (C) Subgroup analysis in handgrip strength over time for studies that used Newton unit. (D) Subgroup analysis in handgrip strength over time for studies that used mmHg unit. (E) Subgroup analysis in handgrip strength over time for studies that used kPa unit. I², heterogeneity between studies; Random, random effects model.

Overall, HGS showed a statistically significant but slight increase in patients with RA over time, with an SMD of 0.25 (95% CI 0.07 to 0.43; p=0.006) and high heterogeneity (I²=86%) (figure 2A). In subgroup analyses, a significant increase in HGS was observed in studies using newtons (N) (mean difference (MD) 30.18 (95% CI 9.29 to 51.08); p=0.005), with moderate heterogeneity (I²=67%) (figure 1C). Similarly, a significant increase was noted in studies using kilopascals (kPa) (MD 9.28 (95% CI 5.26 to 13.29); p<0.001), with low heterogeneity (I²=0%) (figure 1E). On the other hand, no significant changes in HGS were observed in studies using kilograms (kg) (MD −0.33 (95% CI −1.45 to 0.80); p=0.57), with low heterogeneity (I²=0%) (figure 2B). Likewise, no significant changes were found in studies using millimetres of mercury (mm Hg) (MD 24.72 (95% CI −0.80 to 50.24); p=0.06), although high heterogeneity was present (I²=94%) (figure 2D). When studies reporting HGS in N and kg were combined, no significant changes were observed over time (p=0.08; I²=69%), with an absolute MD of 18.67. After converting N into kilograms, the statistical findings remained unchanged (p=0.08; I²=69%), with a MD of 1.31 kg (95% CI −0.18 to 2.80). Conversely, when values expressed in mm Hg and kPa were combined, a significant improvement in HGS over time was observed (p=0.03; I²=89%), with an absolute MD of 25.39 (online supplemental data 7).

**Figure 3:**
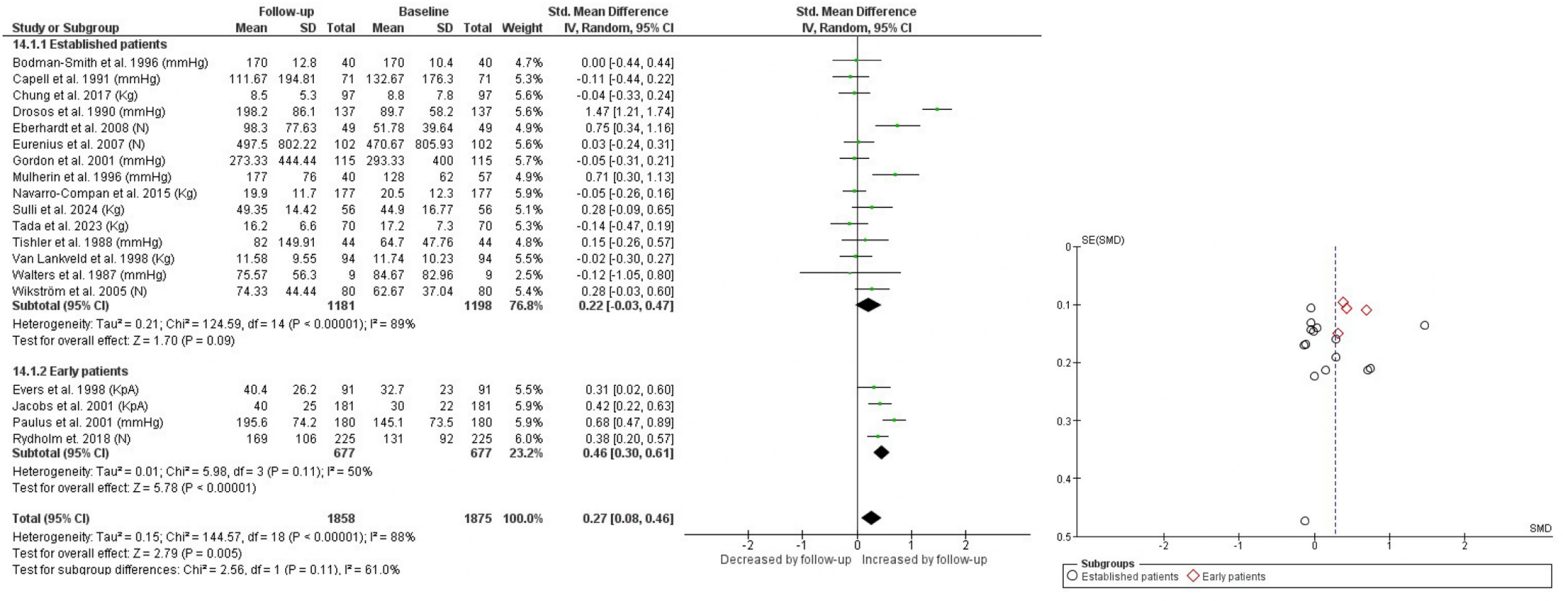
Handgrip strength over time in established patients and early patients. I², heterogeneity between studies. Random, random effects model.

Regarding disease status, patients with early RA (disease duration <1 year) showed an increase in HGS over time, with an SMD of 0.46 (95% CI 0.30 to 0.61; p<0.001) and moderate heterogeneity (I²=50%). In contrast, patients with established RA (disease duration >1 year) showed no significant change in HGS over time (SMD 0.22 (95% CI −0.03 to 0.47); p=0.09), with high heterogeneity (I²=89%) (figure 3). In patients with established RA, when combining studies reporting values in kg and N, no significant changes were observed (p=0.21; I²=60%), with an absolute MD of 15.96. After converting N into kg, results remained non-significant (p=0.25; I²=58%), with an MD of 0.84 kg (95% CI −0.59 to 2.28). Similarly, when considering only studies reporting values in mm Hg, no significant changes were found (p=0.36; I²=96%), with an MD of 22.68 mm Hg (95% CI −25.87 to 71.23). In patients with early RA, combining studies reporting values in kPa and mm Hg showed a significant increase in HGS over time (p<0.00001; I²=60%), with an absolute MD of 18.57. When analysing studies using only kPa, the improvement remained significant (p<0.00001; I²=0%), with an MD of 9.28 KpA (95% CI 5.26 to 13.29) (online supplemental data 7, DOI: 10.17605/OSF.IO/B5P39).

**Figure 4:**
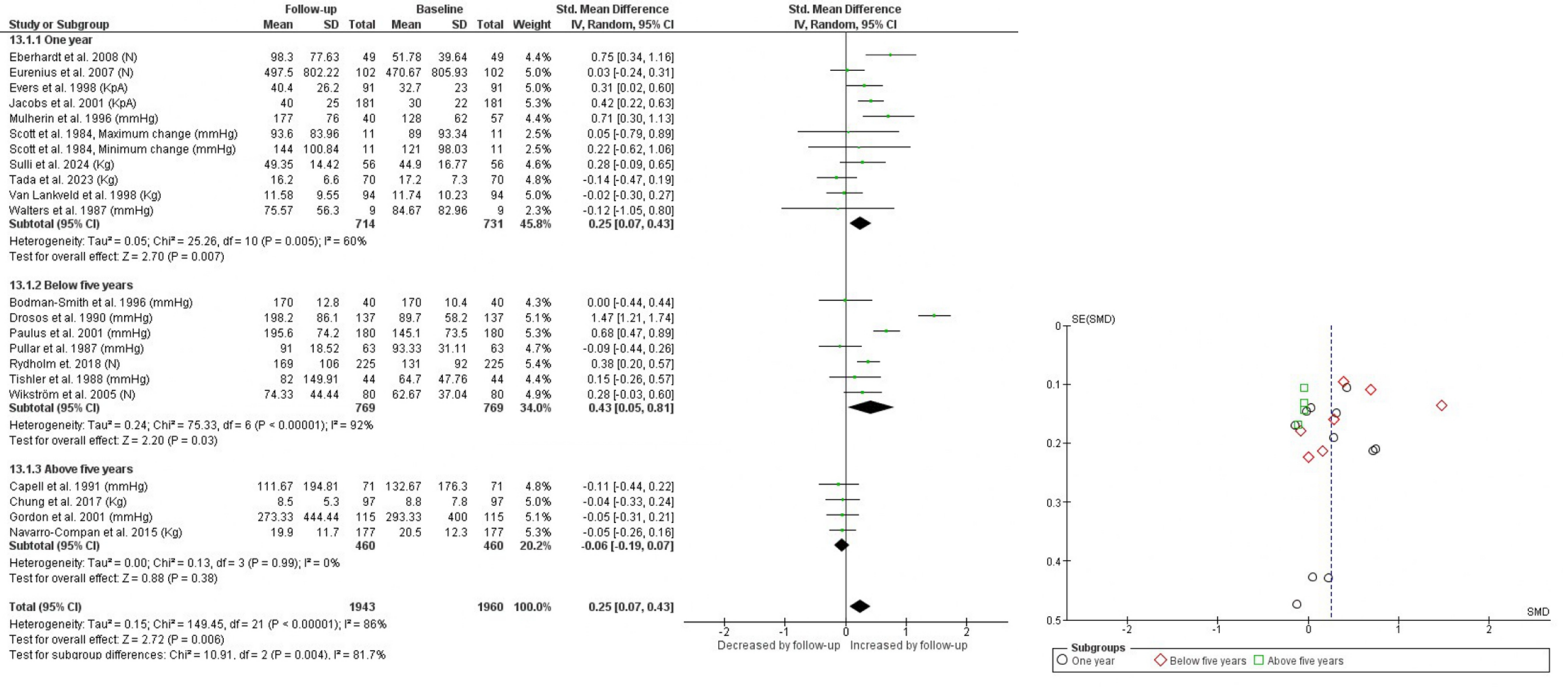
Handgrip strength over time according with time of follow-up. I², heterogeneity between studies; Random, random effects model.

Regarding follow-up duration, patients followed for up to 1 year showed an increase in HGS over time (SMD 0.25 (95% CI 0.07 to 0.43); p=0.007), with moderate heterogeneity (I² = 50%). Similarly, those followed for more than 1 year but less than 5 years also showed an increase (SMD 0.43 (95% CI 0.05 to 0.81); p=0.03), although with high heterogeneity (I² = 92%). Conversely, patients followed for more than 5 years showed no change in HGS (SMD −0.06 (95% CI −0.19 to 0.07); p=0.38), with low heterogeneity (I²=0%) (figure 4). In patients followed for up to 1 year, combining studies with values in kg and N showed no significant change over time (p=0.25; I²=70%), with an absolute MD of 31.68. After converting N into kg, results remained non-significant (p=0.27; I²=70%), with an MD of 1.72 kg (95% CI −1.30 to 4.74). Conversely, when combining studies with kPa and mm Hg, a significant increase in HGS was observed (p<0.00001; I²=0%), with an absolute MD of 16.84 (online supplemental data 7, DOI: 10.17605/OSF.IO/B5P39). In patients followed for less than 5 years, studies reporting mm Hg demonstrated a significant increase (p=0.05; I²=98%), with an MD of 35.04 mm Hg (95% CI 0.60 to 69.49). Studies reporting N showed a similar tendency, although without statistical significance (p=0.07; I²=81%), with an MD of 23.96 N (95% CI −1.79 to 49.72). In patients followed for more than 5 years, no significant changes were observed in studies reporting either kg (p=0.59; I²=0%), with an MD of −0.41 kg (95% CI −1.91 to 1.09), or mm Hg (p=0.45; I²=0%), with an MD of −20.76 mm Hg (95% CI −74.10 to 32.58).

Finally, when changes in HGS were analysed by sex, neither women (SMD −0.09 (95% CI −0.50 to 0.33); p=0.68, high heterogeneity (I²=90%)) nor men (SMD 0.26 (95% CI −0.10 to 0.63); p=0.16, moderate heterogeneity (I²=64%)) showed significant changes over time (online supplemental data 8). After performing the adjustment using the weighted baseline SD, findings remained unchanged over time. In men, no significant changes were observed (p=0.16; I²=64%), with an absolute MD of 13.03. After converting N into Kg, the results also remained non-significant (p=0.13; I²=61%), with an MD of 3.13 kg (95% CI −0.87 to 7.13). Similarly, in women, no significant changes were found (p=0.68; I² = 90%), with an absolute MD of −4.14. After converting N into Kg, statistical findings remained unaltered (p=0.72; I²=91%), with an MD of −0.57 kg (95% CI −3.71 to 2.56) (online supplemental data 7, DOI: 10.17605/OSF.IO/B5P39). The findings indicate that, overall, HGS slightly but significantly increases over time in patients with RA. However, this improvement appears to be influenced by factors such as measurement units, disease duration and follow-up length. The high heterogeneity across several subgroups, along with variations in measurement methods and sample sizes, suggests that these results should be interpreted with caution. Regarding publication bias assessed by funnel plots, all graphs showed symmetry, suggesting no evidence of publication bias.

#### Associations with HGS over time

Among the 27 studies included, 15 evaluated the association between HGS and clinical features or survival. Three studies reported an inverse association between changes in HGS and changes in disease activity over time.^18 19 22^ Two studies^19 22^ also found an inverse association between changes in HGS and physical function. Four studies identified an inverse association between changes in HGS and joint damage^21^ or radiographic changes.^32 34 40^ One reported a positive association between changes in HGS and improvements in self-estimated hand function and the patient’s ability to perform everyday movements.^28^ Another study found that passive pain-coping strategies, such as resting and worrying, predicted changes in HGS.^29^ Only one study reported an association between changes in HGS and survival.^31^ On the other hand, six studies did not find associations between changes in HGS and therapy switch,^34^ changes in joint radiograph scores,^39^ changes in hand disability,^16^ activities of daily living or exercise changes,^17^ change in active leisure^24^ and high physical activity or Good General Health Perception.^36^ Therefore, changes in HGS over time in patients with RA appear to be inversely associated with disease activity, physical function and joint damage. More details are described in table 1 .

**Table 1:** Muscle strength data and its association

First author	Year	Muscle strength equipment	Unit	At baseline	At the end follow-up		Associations
				Total (T) Women (W) Men (M)	Total (T) Women (W) Men (M)	P	
Sulli^16^	2024	An analogic dynamometer (Smedley Dynamometer, Gima, Gessate, Italy)	kg	T: 44.9±16.77	T: 49.35±14.42	0.14	Grip strength change not associated with variation in Hand Test System glove parameters.
Tada^17^	2023	Digital hand-held isokinetic dynamometer (TKK-5401; Takei Scientific Instruments, Niigata, Japan)	kg	T: 17.2±7.3	T: 16.2±6.6	0.026	Grip strength change not associated with activities of daily living or exercise changes
Santo^18^	2020	Handheld dynamometer (Jamar Hydraulic Hand Dynamometer, Preston, USA)	kg	W:14.7±8.3 M:28.5±14.3	W:8.0±68.3 M:23.0±13.3	<0.05	Patients with low disease activity and patients with moderate/high disease activity lost muscle strength after 12 months (p<0.05). However, patients with low disease activity showed higher muscle strength than patients with moderate/high disease activity at both time point (p<0.05); bDMARD use not associated with muscle strength change.
Rydholm^19^	2018	Grippit device (AB Detektor)	N	T:131.0±92.0 W:107.0±73.0 M:185.0±108.0	T:169.0±106.0 W:132.0±104.0 M: 260.0±118.0	NR	The change in DAS28 and the change in HAQ DI explained 26% of the variance in the change in average grip force.
Chung^20^	2017	NR	Kg	T: 8.8±7.8	T: 8.5±5.3	>0.05	No associations evaluated.
Navarro-Compán^21^	2015	Hand-held dynamometer (JAMAR Technologies, Hatfield, PA, USA)	Kg	T: 20.5±12.3 W: 15.6±7.6 M: 33.8±12.8	T: 19.9±11.7 W: 15.7±7.8 M:33.1±13.2	NR	Negative association between joint damage(Joint space narrowing - JSN (B= - 0.087, 95% CI=−0.151 to –0.022,p<0.05), MCP (B= - 0.288, 95% CI=−0.556 to –0.019,p<0.05) and JSN in wrist (B= - 0.132, 95% CI=−0.234 to –0.030,p<0.05) and grip strength; stronger effect in men.
Hallert^22^	2012	Grippit device (AB Detektor)	N	W:89.0±65.0 M: 191.0±116.0	W:108.0±63.0 M:247.0±83.0	NR	HAQ(GEE - 0.002 (95% CI: −0.003 to –0.002), p<0.001)and DAS28(GEE −0.003 (95% CI: −0.0050 to –0.002) p<0.001)predicted muscle strength variation.
Eberhardt^23^	2008	Grippit device (AB Detektor)	N	T: 51.78±39.64	T: 98.30±77.63	<0.001	No association evaluated.
Eurenius^36^	2007	Grippit device (AB Detektor)	N	T: 284 (20–118)	T: 321.5 (44–1127)	0.027	High grip strength was not predicted of high physical activity or Good General Health Perception
Wikström^24^	2005	Grippit device (AB Detektor)	N	T: 52.0 (43.0–93.0)	T: 69.0 (42.0–112.0)	<0.001	Grip strength was not correlated with change in active leisure.
Paulus^25^	2001	NR	mmHg	T: 145.1± 73.5	T: 195.6±74.2	NR	No associations evaluated.
Jacobs^26^	2001	Martin vigorimeter	KPa	T: 30.0±22.0	T: 40.0±25.0	<0.001	No associations evaluated.
Gordon^27^	2001	Inflated bag attached to a sphygmomanometer pressure gauge	mmHg	T: 220 (60–600) (Muscle strength analysis, n=115)	T: 220 (0–600) (Muscle strength analysis, n=115)	0.96	No associations evaluated.
Dellhag^28^	1999	Grippit device (AB Detektor)	N	W: 73.29±61.62 M:174.4±84.78	W: 63.43±50.1 M: 193.87±115.48	>0.05	Maximal grip strength of the dominant hand changes was associated with changes in self-estimated hand function (rs=0.353;p<0.05) and Keitel function test (rs=0.452;p<0.01).
Evers^29^	1998	Martin vigorimeter	KPa	T: 32.7±23.0	T: 40.4±26.2	0.000	Sex (being female) contributed 2% to a decrease in grip strength (Fchange=4.96, p<0.05), the passive pain-coping strategies resting and worrying explained 3% of the variance to change in grip strength (Fchange=3.99, p<0.05)
Van Lankveld^30^	1998	Jamar dynamometer	Kg	T: 11.74±10.23	T: 11.58±9.55	>0.05	Grip strength changes was associated with dexterity changes (r=0.24; 95% CI=0.04, 0.42;p<0.05)
Callahan^31^	1997	NR	mmHg	T: 109.8 SD not reported	T: 82.0 SD not reported	NR	Lower grip strength associated with lower 5 year survival.
Mulherin^32^	1996	Anaeroid dynamometer inflated to 30 mm Hg	mmHg	T: 128.0±62.0	T: 177.0±76.0	<0.001	Grip strength changes was negatively associated with radiographic changes (r=−0.41;p<0.03)
Bodman-Smith^33^	1996	NR	mmHg	T: 170.0±10.4	T: 170.0±12.8	NR	No association evaluated.
Capell^34^	1991	NR	mmHg	T: 94 (33–271) (Muscle strength analysis, n=71)	T: 72 (0–263) (Muscle strength analysis, n=71)	>0.05	Grip strength change not associated with therapy switch; however, Grip strength was shown to correlate well with hand radiograph score at years 1 (r=−0.47) and 10 (r=−0.53;p<0.0005) but not at year 0 .
Drosos^35^	1990	NR	mmHg	T: 89.7±58.2	T: 198.2 ±86.1	<0.001	No association evaluated.
Tishler^37^	1988	NR	mmHg	T: 64.7±7.2 (SE)	T: 82.0±22.6 (SE)	NR	No associations evaluated.
Walters^38^	1987	NR	mmHg	T: 80 (31–143)	T:78.7 (36–112)	>0.05	The authors describe that the immunohistological changes correlate with improvements in grip strength; however, they did not show the data
Pullar^39^	1987	NR	mmHg	T: 88 (75–117)	T: 88 (80–105)	p<0.05	No significant correlation was seen between changes in grip strength and changes in either small or large joint radiograph scores at one or 2 years (Spearman-Rank test; p>0.05).
Scott^40^	1984	standard sphygmonometer cuff inflated to 30 mmHg	mmHg	MAX-C (n=11):89.0±19.9 (SEM) MIN-C (n=11): 121.0±20.9 (SEM)	MAX-C (n=11):93.6±17.9 (SEM) MIN-C (n=11): 144.0±21.5 (SEM)	NR	Relationship of radiological assessment and grip strength (r=−0.27; p<0.05)
Pincus^41^	1984	Standard blood pressure cuff inflated to 30 mmHg	mmHg	T: 113.7 SD not reported	T: 65.5 SD not reported	NR	No associations evaluated.
Million^42^	1984	NR	mmHg	~370*	~450*	NR	No associations evaluated.

* Data extrapolated from IMAGE J

kg, kilograms; KPa, kilopascal; MAX-C, maximum change group; MIN-C, minimum change group; mmHg, millimetres of mercury; N, Newton; NR, not reported.

## Discussion

Our meta-analysis demonstrated a slight increase in HGS over time in patients with RA, particularly among those with early-stage disease or follow-up durations of less than 5 years. In contrast, patients with established RA or follow-up exceeding 5 years showed no significant changes in HGS.

These findings contrast with our initial hypothesis of a decline in HGS over time in individuals with RA. While the observed improvements were not clinically meaningful (<6.5 kg),^43^ they suggest that muscle function may be maintained or even slightly enhanced in many patients with RA. In contrast to the cross-sectional review by Bernanke *et al*,^7^ which reported stable HGS across individuals aged 35 to 65 years, our longitudinal analysis reveals intraindividual improvements over time—particularly among those with early-stage disease or shorter follow-up periods (<5 years). Methodological differences, including study design and population characteristics, likely account for these divergent findings.

Most studies in this review included patients with RA treated with MTX (a csDMARD), with only one study involving bDMARDs, and all patients were followed in outpatient clinics. While pharmacological treatments aim to reduce disease activity and organ involvement, evidence on their effects on HGS is limited. We speculated that pharmacological treatment affects HGS in two ways: first, treatments for RA attenuate disease activity by blocking inflammatory mediators and their signalling or by inducing anti-inflammatory and regulatory pathways; second, indirectly, the decrease of inflammatory mediators leads to regulation in muscle tissue. This indicates a dual effect on both clinical outcomes and physical function in these individuals.^44 45^ However, this review did not account for physical activity or exercise interventions, which may also influence HGS outcomes.

Regarding disease status, our findings showed that patients with early RA experienced increases in HGS over time, while those with established RA did not. This may be related to better inflammatory control early in the disease. Moradi *et al*^46^ reported muscle dysfunction even in preclinical RA, while Lemmey *et al*^44^ found higher HGS in patients in remission. Thus, improvements in HGS may occur primarily in early RA, driven by treatment response, whereas the chronicity of established RA may limit such gains despite disease control.

Regarding follow-up duration, we found that patients followed for up to 5 years showed increases in HGS, while those followed for more than 5 years did not. A long-term cohort study of patients with RA reported clinically meaningful reductions in disease activity and improvements in physical function among individuals with established RA between 2005 and 2014.^47^ In line with our findings, this initial improvement may reflect effective disease management and reduced inflammation, whereas the plateau observed after 5 years could be attributed to RA progression, long-term medication effects or age-related muscle decline. Notably, the absence of HGS deterioration during long-term follow-up suggests preserved muscle function, likely due to sustained disease control. Furthermore, in studies with follow-up periods exceeding 5 years, the lack of continued improvement in HGS may reflect age-related declines in muscle mass and function, rather than disease-specific trajectories alone.

Interestingly, when adjustments were performed by grouping units of measurement, changes over time appeared more evident in studies using mm Hg and kPa compared with those using kg or N. This finding may be related to the greater sensitivity of devices based on pressure measures, which are able to capture more subtle variations in HGS over time.

These findings may reflect associations reported in the included studies, as HGS is linked to disease activity, joint damage, physical function and survival. Previous meta-analyses show that low HGS is associated with higher risk of knee osteoarthritis, heart failure complications, disability, cognitive and mobility decline in older adults, and increased mortality across healthy, elderly and clinical populations.^48^,^54^ This may relate to the endocrine role of muscle tissue, which secretes myokines such as myostatin, irisin and IL-6.^55^ Muscle-derived myokines circulating in the serum can have both beneficial and harmful effects in RA, with their impact varying according to the type of myokine, its cellular source and the broader inflammatory context.^56^,^58^ The deregulation of muscle tissue and exacerbation of myokines to serum can lead to a decrease in HGS and deregulation of systems such as bone and endocrine. Consequently, these changes may lead to worsening clinical features in RA.

This is the first systematic review to focus on longitudinal changes in HGS in RA, addressing an important gap in the literature. By including only prospective studies, we could evaluate changes over time rather than cross-sectional differences. On the other hand, several limitations should be considered. The evidence included in this review showed substantial heterogeneity in methods, assessment tools and follow-up durations, with few studies using standardised units like Newtons or kilopascals and most relying on kilograms, complicating comparisons. Many studies lacked data on physical activity, a key confounder in muscle strength analyses, and few had long-term follow-up or were powered to assess sex-specific trends. Despite these limitations, the review process followed a rigorous protocol, including comprehensive searches, dual independent screening using Rayyan and manual data extraction, enhancing reliability. However, sensitivity analyses and the Grading of Recommendations Assessment, Development, and Evaluation (GRADE) assessments were not performed due to the descriptive nature and heterogeneity of the data, and the overall low methodological quality of included studies may limit generalisability. These findings have several implications: future research should focus on long-term longitudinal studies, stratify by disease duration and treatment and assess the effect of targeted strength interventions. Clinicians should prioritise early HGS interventions, educate patients on physical activity and incorporate regular HGS monitoring into care. At the policy level, integrating HGS assessments into rheumatology guidelines, promoting early screening for muscle weakness and developing physical activity recommendations tailored for patients with RA are essential to support functional outcomes and disease management.

## Conclusion

In conclusion, the stage of disease onset and the duration of follow-up are critical factors influencing longitudinal changes in HGS among patients with RA. Our findings indicate that individuals with early-stage RA tend to exhibit improvements in HGS over time, whereas those with established RA or a longer disease duration demonstrate more stable strength levels. However, the generally poor methodological quality of most studies and the high heterogeneity observed necessitates caution when interpreting these results. Nonetheless, the findings underscore the potential benefits of early interventions aimed at enhancing HGS in patients with RA as well as the importance of regular monitoring to mitigate disease-related physical decline. Future studies need to standardise the assessment methods and units used to measure HGS. Such standardisation would increase comparability between studies and improve the clinical interpretability of findings.

## Supplementary material

10.1136/bmjsem-2025-002617 online supplemental file 1

10.1136/bmjsem-2025-002617 online supplemental file 2

10.1136/bmjsem-2025-002617 online supplemental file 3

10.1136/bmjsem-2025-002617 online supplemental file 4

10.1136/bmjsem-2025-002617 online supplemental file 5

10.1136/bmjsem-2025-002617 online supplemental file 6

10.1136/bmjsem-2025-002617 online supplemental file 7

10.1136/bmjsem-2025-002617 online supplemental file 8

## Data Availability

All data relevant to the study are included in the article or uploaded as supplementary information.
