# Supplementary material for: Longitudinal monitoring of handgrip strength in rheumatoid arthritis: a window into for disease activity—a systematic review with meta-analysis
Source: BMJ Open Sport Exerc Med. 2025 Nov 21;11(4):e002617. doi: 10.1136/bmjsem-2025-002617 (PMC12658546; doi:10.1136/bmjsem-2025-002617)
Supplement: online supplemental file 4 [file bmjsem-11-4-s004.pdf]

Supplemental Data 4. Patients' baseline characteristics

First author	Year	Country	Time of follow-up	Total Sample	Women	Men	Age (years old)	Disease duration (years)	Disease activity methods	Disease Activity Value	Physical function methods	Physical function Value
Sulli[16]	2024	Italy	7±2 months	56	51	5	61 ± 13	13 ± 8	DAS28-CRP	2.87 ± 1.31	HAQ-DI	7.5 (7.5 – 21.25)
Tada[17]	2023	Japan	1 year	70	57	13	66 (58.8 -73.0)	6.5 (1.4 -11.7)	DAS28-ESR	3.05 ± 0.88	mHAQ	0.25 (0 – 0.725)
Santo[18]	2020	Brazil	1 year	90	78	12	56.5 ± 7.3	8.5 (3.0–18.0)	DAS28-CRP	3.0 (1.0–3.0)	HAQ-DI	1.2 ± 0.1
Rydholm[19]	2018	Sweden	5 years	225	159	66	60.0 ± 14.7	7 (5–10) months	DAS28	4.6 ± 1.4	HAQ	0.85 ± 0.62
Chung[20]	2017	USA/Canada/Uk	7 years	97[a]	64	33	61.8 ± 10.5	NR	NR	NR	NR	NR
Navarro-Compán[21]	2015	Norway	10 years	177	129	48	50.5 ± 12.8	2.2 ± 1.2	Ritchie articular index	9.5 ± 6.1	HAQ	0.90 ± 0.62
Hallert[22]	2012	Sweden	8 years	149	101	48	W: 55.0 ± 16.0<br>M: 60.0 ± 14.0	≤ 12 months	DAS28-ESR	W: 5.3 ± 1.2<br>M:5.4 ± 1.1	HAQ	W: 0.9±0.6<br>M:0.8±0.6
Eberhardt[23]	2008	Sweden	1 year	49	37	12	54.9 ± 12.2	15.0 ± 10.0	DAS28-ESR	6.06 ± 1.08	HAQ	1.55 ± 0.57
Eurenius[36]	2007	Sweden	1 year	102	76	26	57 (19-84)	15 (4-48) months	DAS28	3.46 (0–7.21)	HAQ	0.57 (0–2.38)
Wikström[24]	2005	Sweden	2.8 (2.0-3.4) years	80	66	14	54.0 (47.0-66.0)	4.0 (1.6–11.2)	Global assessment of disease activity	4.6 (2.8–6.4) cm	HAQ	1.2 (0.7 – 1.6)
Paulus[25]	2001	USA	2 years	180	141	39	51.5 ± 12.8	6.0 ± 3.4 months	DAS28-ESR	4.92 ± 1.14	HAQ	1.22 ± 0.73
Jacobs[26]	2001	Netherlands	1 year	181[b]	NR	NR	57.0 ± 14.0	recent onset RA	Joint score	142 ± 100	NR	NR
Gordon[27]	2001	UK	10 years	289	210	79	59 (19–82)	10 (1–61) years	Ritchie articular index	5.0 (0–23)	HAQ	13 (0–60)
Dellhag[28]	1999	Sweden	5 years	43	28	15	W:52.7 (29–69)<br>M: 55.5 (41–69)	W:7.5 (6–10)<br>M: 7.8 (6.10)	NR	NR	HAQ	W: 1.13±0.56<br>M: 0.75±1.09
Evers[29]	1998	Netherlands	1 year	91	64	27	57 (20-82)	Recently diagnosed RA	Joint score	96.9 ± 95.9	NR	NR
Van Lankveld[30]	1998	Netherlands	1 year	94	59	35	56.34 (20-78)	13.3 (1-47)	NR	NR	NR	NR
Callahan[31]	1997	USA	5 years	210	105	105	56.6	9.7	NR	NR	mHAQ	2.0
Mulherin[32]	1996	Ireland	1 year	40	28	12	46.4 ± 13.3	2.4 ± 2.9	Ritchie articular index	16.0 ± 12.0	NR	NR
Bodman-Smith[33]	1996	UK	4 years	40[c]	26	14	W: 52.5<br>M:50.4	2 years of the onset of RA (76% within 1.5 years)	Joint score	9.6±1.17	NR	NR
Capell[34]	1991	UK	10 years	92	74	18	49 (21-71)	5 (1-27)	Ritchie articular index	33 (4-58)	NR	NR
Drosos[35]	1990	Greece	2 years	137	103	34	56.05 ± 12.67	8.76 ±5.5	NR	NR	NR	NR

Tishler[37]	1988	Israel	3 years	44	41	3	60.2 (30-87)	10.5 (1-33)	Ritchie articular index	35.0±2.0	NR	NR
Walters[38]	1987	UK	6 months	9[d]	5	4	64 (45-76)	11.1 (2.4-26)	Modified Ritchie articular index (RAI).	27 (17-44)	NR	NR
Pullar[39]	1987	Scotland	2 years	63	NR	NR	NR	NR	Ritchic articular indcx	17 (12-22)	NR	NR
Scott[40]	1984	UK	1 years	22[e]	NR	NR	NR	NR	NR	NR	NR	NR
Pincus[41]	1984	USA	9 years	75	53	22	54.7 (27-79)	11.2 (2-32)	NR	NR	NR	NR
Million[42]	1984	UK	10 years	37[f]	29	8	48.0±11.7	Definite or classical rheumatoid arthritis' for 2-24 months	NR	NR	NR	NR

NR, not reported; W, Women; M, Men; UK, the United Kingdon; USA, The United States

[a] Only Non silicone metacarpophalangeal joint arthroplasty group

[b] Only DMARD treatment group

[c] We select a cohort with more patients - Cohort A

[d] Only group 2

[e] 64 patients were included; however, only 22 patients that showed changes in the Larsen index was analysed to grip strength

[f] Only Active steroid (AS) group

* SD not reported
