# Supplementary material for: Longitudinal monitoring of handgrip strength in rheumatoid arthritis: a window into for disease activity—a systematic review with meta-analysis
Source: BMJ Open Sport Exerc Med. 2025 Nov 21;11(4):e002617. doi: 10.1136/bmjsem-2025-002617 (PMC12658546; doi:10.1136/bmjsem-2025-002617)
Supplement: online supplemental file 5 [file bmjsem-11-4-s005.pdf]

Supplemental Data 5. Pharmacological treatments of studies included

First Author	Year	MTX , n (%)	Other DMARDs, n (%)	Biologic, n (%)	Glucocorticoid , n (%)
Sulli[16]	2024	NR[a]	NR [a]	NR[a]	NR[a]
Tada[17]	2023	61 (87.1)	NR	29 (41.4)	15 (21.4)
Santo[18]	2020	52 (57.8)	14 (15.5)	27 (30.0)	53 (58.9)
Rydholm[19]	2018	117 (52)	68 (30)	NR	88 (39)
Chung[20]	2017	NR	NR	NR	NR
Navarro-Compán[21]	2015	NR	93 (52.5)	NR	44 (24.9)
Hallert[22]	2012	62 (41.6)	NR	NR	NR
Eberhardt[23]	2008	NR	NR	49 (100)	NR
Eurenius[36]	2007	NR	86 (84.3)	NR	34 (33.3)
Wikström[24]	2005	NR	74 (92)	NR	28 (35)
Paulus[25]	2001	NR	180 (100)	NR	NR
Jacobs[26]	2001	NR	181 (100)	NR	NR
Gordon[27]	2001	2 (0.7)	98 (33.9)	NR	NR
Dellhag[28]	1999	NR	NR	NR	NR
Evers[29]	1998	23(25)	13 (14)	NR	13 (14)
Van Lankveld[30]	1998	NR	NR	NR	NR
Callahan[31]	1997	NR	NR	NR	NR
Mulherin[32]	1996	16 (40)	27 (67.5)	0 (0)	17 (43)
Bodman-Smith[33]	1996	NR	NR	NR	NR
Capell[34]	1991	14 (15)	NR	NR	7 (8)
Drosos[35]	1990	137 (100)	NR	NR	NR
Tishler[37]	1988	44 (100)	NR	NR	NR
Walters[38]	1987	NR	9 (100)	NR	NR
Pullar[39]	1987	NR	63 (100)	NR	NR
Scott[40]	1984	NR	NR	NR	NR

Pincus[41]	1984	NR	14/52 (27%)	NR	37/53 (70%)[b]
Million[42]	1984	NR	NR	NR	37 (100)

NR, Not reported

[a] The drug taken by patients were not considered neither at entry non during follow-up.

[b] Number of patients who had taken drugltotal who responded to question.
