# Supplementary material for: Longitudinal monitoring of handgrip strength in rheumatoid arthritis: a window into for disease activity—a systematic review with meta-analysis
Source: BMJ Open Sport Exerc Med. 2025 Nov 21;11(4):e002617. doi: 10.1136/bmjsem-2025-002617 (PMC12658546; doi:10.1136/bmjsem-2025-002617)
Supplement: online supplemental file 6 [file bmjsem-11-4-s006.pdf]

Supplemental Data 6. Methodological Quality Assessments of Included Studies

		Selection					Comparability	Outcome					
First Author	Year	1	2	3	4	Total	1	1	2	3	Total	Total	AHRQ
Sulli[16]	2024	1	1	1	0	3	0	1	0	0	1	4	Poor quality
Tada[17]	2023	0	1	1	0	2	0	1	1	1	3	5	Poor quality
Santo[18]	2020	1	1	1	0	3	1	1	1	1	3	7	Good quality
Rydholm[19]	2018	0	1	1	0	2	1	1	1	1	3	6	Fair quality
Chung[20]	2017	1	1	1	0	3	1	1	1	1	3	7	Good quality
Navarro-Compán[21]	2015	0	1	1	0	2	1	1	1	1	3	6	Fair quality
Hallert[22]	2012	0	1	1	0	2	1	1	1	1	3	6	Fair quality
Eberhardt[23]	2008	0	1	1	0	2	0	1	0	0	1	3	Fair quality
Eurenius[36]	2007	1	1	1	0	3	0	1	0	1	2	5	Poor quality
Wikström[24]	2005	0	1	1	0	2	0	1	1	0	2	4	Poor quality
Paulus[25]	2001	0	1	1	0	2	0	1	1	0	2	4	Poor quality
Jacobs[26]	2001	0	1	1	0	2	1	1	1	0	2	5	Fair quality
Gordon[27]	2001	0	1	1	0	2	0	1	1	1	3	5	Poor quality
Dellhag[28]	1999	0	1	1	0	2	1	1	1	0	2	5	Fair quality
Evers[29]	1998	0	1	1	0	2	0	1	1	0	2	4	Poor quality
Van Lankveld[30]	1998	0	1	1	0	2	0	1	1	1	3	5	Poor quality
Callahan[31]	1997	0	1	1	0	2	0	1	1	1	3	5	Poor quality
Mulherin[32]	1996	0	1	1	0	2	0	1	1	1	3	5	Poor quality
Bodman-Smith[33]	1996	0	1	1	0	2	1	1	1	1	3	6	Fair quality
Capell[34]	1991	0	1	1	0	2	1	1	1	1	3	6	Fair quality
Drosos[35]	1990	0	1	1	0	2	0	1	1	0	2	4	Poor quality
Tishler[37]	1988	0	1	1	0	2	0	1	1	0	2	4	Poor quality
Walters[38]	1987	0	1	1	0	2	0	1	0	1	2	4	Poor quality

Pullar[39]	1987	0	1	1	0	2	0	1	1	1	3	5	Poor quality
Scott[40]	1984	0	1	1	0	2	1	1	1	1	3	6	Fair quality
Pincus[41]	1984	0	1	1	0	2	0	1	1	1	3	5	Poor quality
Million[42]	1984	0	1	1	0	2	0	1	1	0	2	4	Poor quality

AHRQ , the Agency for Healthcare Research and Quality standards
