# Supplementary figures and images for: Longitudinal monitoring of handgrip strength in rheumatoid arthritis: a window into for disease activity—a systematic review with meta-analysis
Source: BMJ Open Sport Exerc Med. 2025 Nov 21;11(4):e002617. doi: 10.1136/bmjsem-2025-002617 (PMC12658546; doi:10.1136/bmjsem-2025-002617)

A.

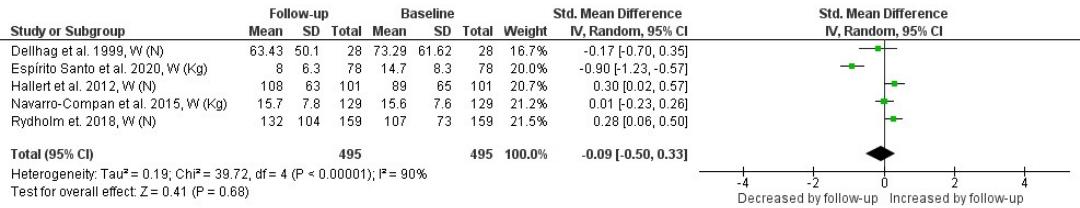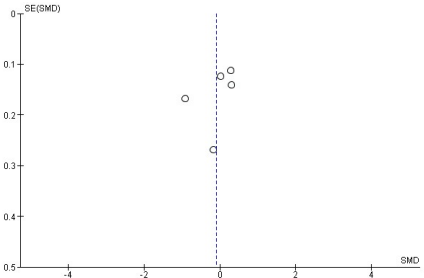

B.

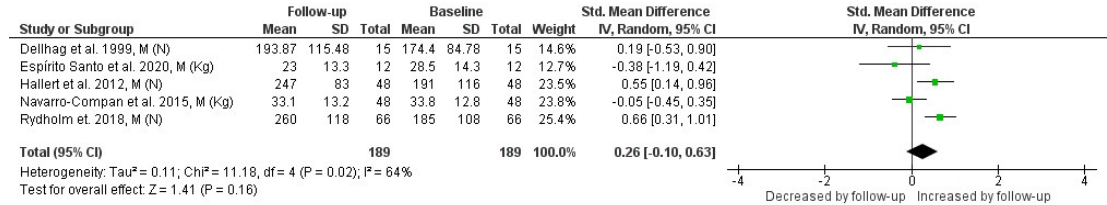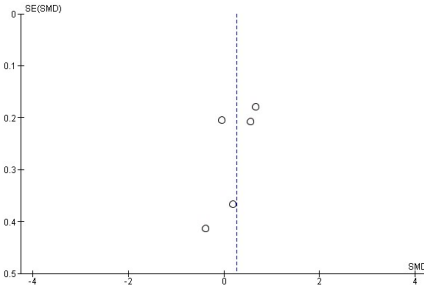

Supplement: online supplemental file 8 [file bmjsem-11-4-s008.pdf]
